# Effects of Women’s Work-Family Multiple Role and Role Combination on Depressive Symptoms in Korea

**DOI:** 10.3390/ijerph17041249

**Published:** 2020-02-15

**Authors:** Ji-won Kang, Soong-nang Jang

**Affiliations:** Red Cross College of Nursing, Chung-Ang University, Seoul 06974, Korea; shkjw0401@gmail.com

**Keywords:** multiple role, depression, role combination, work-family balance

## Abstract

This study set out to investigate the effects of multiple roles on depressive symptoms in women. The role of women was divided into worker, household worker, spouse, parent, and caregiver roles to identify the differences in depressive symptoms according to the number of roles, role-fulfillment, and role-combination. Using the sixth raw data of the 2016 Korean Longitudinal Survey of Women and Families for analysis, the data had 6198 respondents who did not have missing values in the major variables. There are three main findings of this study: (1) as the number of roles increased, depressive symptoms of women was decreased. In addition, role-combination was a more meaningful element; (2) women who did not have any roles tended to be more depressed; (3) the caregiver role showed a negative effect on depressive symptoms of women. This study was to include the various aspects of women’s roles and to determine the effects of multi-roles on depressive symptoms in women.

## 1. Introduction

Most modern women play various forms of multiple roles. According to the Korean Economically Active Population Survey, the proportion of women participating in economic activity in August 2019 was 53.6%, showing a continuously increasing tendency [1]. Married working women experience more negative than positive transitions in work–family transfers compared with men, and women, unlike men, had non-specific and pervasive role-conflict and feel responsible for problems that occur with multiple roles [2,3]. As women advance into society, the burden of social roles other than housework and childcare also expands. These women’s multiple roles may pose a burden to women, such as role conflict, but they also give them the potential to exploit various social resources [4,5,6,7]. Previous studies have noted that role combination was an important factor in explaining the effect of multiple roles on mental health. Based on the results of these studies, it is necessary to focus on the role combination and identify the depression of women [8,9,10,11]. 

Discussions on the impact of women’s multiple roles have developed along the stream of the work–family theory as women have taken jobs away from traditional women’s roles. The theory of work–family balance originates from a segmentation model, in which the work and family are separated and, thus, unaffected. This theory has prompted the emergence of a conflict model, in which demands of roles at home and in the workplace cause disputes and conflicts, and a compensation model, in which one wants to be rewarded for performing other roles. Recently, research on the multi-role of women has focused on the spillover and borderline theories; the former highlights the positive and negative effects of interactions at workplace and home, whereas the latter states that individuals cross the borderline of the workplace and home. Women experience role conflicts at home and at work, which has both positive and negative health effects. The role insufficiency model, which explains the fact that role conflict and problems can arise when different roles require the same resource, has restrictions to explain the negative transitions due to the limited amount of resources. The negative effects of work–family conflict (WFC) and family-work conflict (FWC) are divided into the direction of the negative effects of multiple roles. The role-expansion hypothesis that explains positive transition has been described as work–family enrichment, work–family enhancement, and work–family facilitation. As theories developed, the topics of research have expanded from focusing on only the negative effects of women’s multi-role to exploring its positive effects, which then led to the study of work–family balance [12,13].

The literature of women’s multiple roles and health has demonstrated conflicting results, including both negative and positive effects, and a variety of theories. The first is the view that multi-roles have a negative impact on physical and mental health. According to the analysis results of the employment status of married women with children, the health of employed women was worse [14]. Comparing to that of housewives, the self-rated health of unmarried mothers without a job was poor, depression was high, and married working women were the most stressed [15]. A study on Korean married women found that women rearing children who were involved in simple labor and sales service jobs were heavily influenced by the multi-role burden, and those in child-age families with professional careers were significantly affected by multi-role attachment [16]. A meta-analysis of studies involving work–family conflict, including multiple roles from 1999 to 2006, concluded that work–family conflicts had a negative impact on physical health, depression, and stress [17]. 

By contrast, other studies have suggested that multiple roles have a positive impact on health [5,18]. A study analyzing the health effects of multiple roles by income quartiles indicated that physical and mental health levels were lower for unmarried mothers who did not have a job, and the health of housewives who performed only household work was worse compared with married women with children [19]. A study on health status by age group and number of roles also revealed that middle-aged women had the best health status when they played three or more roles [20]. Additionally, a study on health status by occupation revealed that good health status was found in the following order: non-physical work, housewife, and physical work [21]. Recently, studies on the effects of positive and negative spillover on health found that work–family conflicts had a negative effect on depression and work–family facilitation had a positive effect on depression; these studies were based on the theory that work–family conflict and promotion occur simultaneously [12]. 

Considering Korean women’s social participation is transitional, it is possible that these international results in which multiple roles have a positive effect on health can be applied differently in Korean society. Korean women’s participation in economic activity is below the OECD average and women’s social status is relatively low. Korean women have been facing the precarity of labor, such as low employment rates, high temporary worker rates, low wages, high gender wage differential, M-shaped curves of employment rates, and so on [22,23,24,25]. This gender-precarity nexus occurred in responsibilities in the reproductive sphere that women took on [26]. It was caused by the overlap between the disadvantages as women in the labor market and the drawback of performing maternal roles [27]. In a previous study that addressed the value of work–family balance of married working women in Korea, Japan, and the UK, a lot of Korean women, more than other countries, had an awareness that women had to resign when facing work–family conflict situations [20]. A study comparing the UK and Japan with similar gender values to Korea showed that Japanese women had greater role conflict than British women, and their mental health was relatively poor when they had multiple roles [28]. Another study comparing Finland, Japan, and the UK reported that Japanese women had the greatest conflict and poorest psychological health [21].

The “career interruption women” is a term to explain women who ceased their economic activities due to their maternal role. This term regards maternal labor in areas outside the labor domain as having a loss in women’s labor capacity and represents a perception of the low value of care labor of women [22]. In this context, it is important to distinguish and define women’s various roles in the family sphere and to analyze the impact of women’s multiple roles. The Korean literature on women’s multi-role and health has focused mainly on women with limited roles, such as married women or married women with children. Therefore, research on the effects of multi-role on health is insufficient. An increase in the aged population emphasizes the importance of the role of caregivers [14]. Especially, women in a family typically tend to care for older adults with disabilities or chronic diseases; role strain typically increases when such a caregiver role is added to the multiple roles of women [29,30]. Therefore, expanding the scope of roles to identify various aspects of roles that women play is necessary. We hypothesized that Korean women’s role-related depressive symptoms may be different from previous studies if we analyze a broader range of role types and combinations, including caring roles as independent variables.

This study aimed to investigate the effects of multiple roles on depressive symptoms in women. In this paper, we attempt to broaden women’s roles toward caregiver from traditional roles such as spouse, worker, and parent. We used the role-combination concept to identify key roles influencing women’s depressive symptoms. The roles of women were divided into worker, household worker, spouse, parent, and caregiver to identify the differences in depressive symptoms according to the number of roles, role- fulfillment, and role-combination.

## 2. Methods

### 2.1. Data Collection

Data were collected from 6198 respondents who did not have missing values in the major variables of the sixth raw data of the 2016 Korean Longitudinal Survey of Women and Families (KLoWF). Raw data is open access data, which anyone can access and use through the KLoWF website. The KLoWF was a longitudinal study and used a stratified multistage sampling design using Korean Population and Housing Census data. No consent forms are required, and free download is available after completing the purpose of use. The main survey has been conducted every two years since the first and second surveys in 2007 and 2008, respectively. Computer Assisted Personal Interviewing (CAPI) was used to collect data. Informed consent was obtained for all respondents, which included an explanation of the survey purpose and data usage. The survey included questions related to households, such as type of housing and household income, assets, and liabilities; personal experiences, such as family growth, marital life, childbirth experience, family relations, division of housework, family-related values, health, and aging; and working conditions, such as economic activities, job satisfaction, discrimination, and maternity protection. In the sixth survey, there were 12,285 female household members aged between 19 and 64 years in 9711 households nationwide, excluding Jeju and the other islands.

### 2.2. Measurement Variables

#### 2.2.1. Roles of Women

Women’s roles were divided into worker, household worker, spouse, parent, and caregiver. Respondents who said that they “have a job” in the survey were classified as having a worker role. Having jobs was defined as working for more than one hour at a specific place for an income within a week from the time of the survey or working for their families’ economic activity even if they were not paid. It followed the definition of KLoWF and was the same definition of “employed” in the Korean Economically Active Population Survey of Statistics Korea by the Korean Ministry of Labor. The role of household worker was defined as having a daily average housework time to be above respondents’ average. The parent role was defined as being the main caregivers of a child or grandchild under 19 years of age. The spouse role was defined as being married or in a factual marriage relationship; those who were single, bereaved, divorced, or separated were not classified as having a spouse role. The role of caregiver was defined as being the main caregivers of their parents or parents-in-law in case they needed help because of age or poor health.

#### 2.2.2. Combination of Roles

Women’s five roles were combined and listed in order of frequency and then divided into 11 role groups, top 10 role groups, and the remaining other role groups.

#### 2.2.3. Depressive Symptoms

Depressive symptoms were measured using a 10-item version of the Center for Epidemiologic Studies Depression scale (CES-D), calculated as the sum of responses to 10 questions [31]. The response for each item was 4-point Likert scale (range from 0 to 3). In the KLoWF, the CES-D 10 has values ranging from 0 to 30, with a score of 30 indicating the most severe depressive symptoms. This Korean version of CES-D 10 has been reported as valid in previous studies [32,33].

#### 2.2.4. Diagnostic Disease

Diagnostic disease was defined as having more than one disease currently being diagnosed by a doctor. Regarding the question about diagnostic disease in the questionnaire, it was defined that those who responded by being diagnosed with one or more diseases by a doctor had a diagnostic disease, and the others was ‘none’. 

#### 2.2.5. Income

Income was included in the analysis by dividing the household gross income into quartiles over the past one year. Last year’s gross household income was the pretax income of all members of the household, which included not only earned and business incomes but also financial and real estate incomes.

### 2.3. Analysis

Data analysis was performed using SPSS 25 (SPSS Corp., Armonk, NY, USA). Demographic, role, and health-related variables of women were analyzed using descriptive statistics. A simple regression analysis was performed to evaluate the differences in severity of depressive symptoms by demographics, health status, and multiple roles. The effects of role-fulfillment, number of roles, and role combinations of women were analyzed using a multiple regression analysis.

## 3. Results

### 3.1. Demographics

Participants’ mean age was 53.01 years. The skewness of age variable is −0.001 and kurtosis is −1.038. The most frequently appeared education level was high school at 36% (2229 participants) (Table 1). The mean of income was KRW 42.4935 million. Most were married (83.1%, 5150 participants). About 29.8% of participants were diagnosed with a disease, and diagnosis with one type of disease was the most common (24.2%). The mean score of CES-D10 was 4.35 (skewness = 1.443, kurtosis = 2.119); 15.2% of participants had depression scores of 10 or more. The proportion of females with roles of workers was 60.5%; houseworker, 52%; parenting, 40.7%; spouses, 83.1%; and caregivers, 1.1%. The most common number of roles was three (35%). The most common combination was the (worker-spouse-houseworker) group (14%).

### 3.2. Effects of Multi-Role on Depressive Symptoms in Women

Regarding the effect of women’s role performance on depressive symptoms, the results of a multiple regression analysis showed that roles of worker (B = −0.521, *p* = 0.000), houseworker (B = −1.611, *p* = 0.000), and spouse (B = −0.673, *p* = 0.000) had a positive effect on depressive symptoms, whereas that of caregiver (B = 1.379, *p* = 0.015), a negative effect (Table 2). Higher income (B= −0.396, *p* = 0.000) was less depressive. Participants with an educational level higher than college (B = −0.485, *p* = 0.048) were less depressed than those who were below elementary school. Depressive symptoms were more severe in the presence of a diagnosed disease (B = 2.662, *p* = 0.000). Age and parent role were not statistically significant.

### 3.3. Effects of the Number of Women’s Roles on Depressive Symptoms

The results of a multiple regression analysis for the effect of the number of roles played by women on depressive symptoms showed that the CES-D10 score tended to decrease as the number of played roles increased (B = −0.647, *p* = 0.000). Age and income were negatively associated with depressive symptoms. Those who were below the college education tended to be less depressed (Table 3).

### 3.4. Effects of Women’s Role Combination on Depressive Symptoms

A multiple regression analysis was performed to determine the effect of role combinations on depressive symptoms. Compared with the (None) groups, other role groups showed less depressive symptoms. The least depressed group was (Worker-Spouse-Houseworker) (B = −1.351, *p* = 0.000), followed by (Parent-Spouse-House worker) (B = −3.324, *p* = 0.000), (Parent-Worker-Spouse-Houseworker) (B = −3.305, *p* = 0.000), (Spouse-House worker) (B = −2.855, *p* = 0.000), and (Worker-Spouse) (B = −2.839, *p* = 0.000). Income level (B = −0.418, *p* = 0.000) and diagnostic disease (B = 2.629, *p* = 0.000) was negatively and positively associated with depressive symptoms, respectively. The above college graduate group was significantly less depressed than the primary education graduate group (B = −0.493, *p* = 0.045). Age was not statistically significant (Table 4).

## 4. Discussion

This study was a cross-sectional survey to identify the effects of multiple role performance and role combinations on depressive symptoms in women. Women’s roles were divided into worker, houseworker, spouse, parent, and caregiver. Specifically, the effects of the type, number, and combinations of roles on depressive symptoms were analyzed. The results showed that women’s multi-role performance could have positive effects on depressive symptoms. This section discusses the three main findings of this study.

First, as the number of roles increased, women tended to be less depressed. This finding supports the role expansion theory that multi-role is positive for health and previous findings stating that performing a multi-role is positive for mental health [34]. Longitudinal studies of Swedish women showed that mental health is better with increasing social roles defined as parent, spouse, and worker roles [35]. Because performing a role creates energy to endure other roles, and the resources from the products of social relations can be used for other roles, experiences from one role can produce positive experiences and results from other roles [34,36,37]. However, there is a limit in the interpretation of this study in that simply increasing the number of roles leads to less depression. This is because the caregiver role has a negative effect on depressive symptoms. When an additional regression analysis was performed for each number of roles as dummy variables with reference to the group with zero role counts, the group with five role counts had no statistical significance. The explanation here could be because the five-role group included the caregiver role, which was negatively associated with depressive symptoms. This finding suggests that a combination of roles is more important than simply accumulating roles in depressive symptoms of women. Although the accumulation of roles has a positive effect on women’s mental health, their effects can be offset by the types of roles they play. There was no evidence that the effect of multiple roles of Korean women on depressive symptoms differed from other countries, contrary to expectation. These days, the Korean government tries to improve the work environment in which employees can balance work and family. They compel the employer to provide maternity leave and allowances or working hour reduction for women who raise children under nine years old. Also, they encourage the father to use paternity leave, offering high allowances, which are 100% of their ordinary wage. In addition, they try to increase daycare centers in the working place in order to reduce work–family burden. While there appears to be no definitive evidence about the effect of these policies, it seems reasonable to assume that work–family balance policies have an effect on the reduction of role conflict of women. Notwithstanding the Korean government’s effort, in part, a practical problem still remained in Korean women aged 30–40s. According to Statistics, employment retention rate of paternity leave users was 79.1%, the others resigned or were fired in 2017 [38]. Considering this social context, the social system should be supported more so that women can maintain their roles in various areas. Furthermore, it will be necessary to change the perception that women’s roles in the family sphere make women cut off their career in order to re-evaluate the value of women’s roles. In this study, there were limitations in applying the same characteristics of each type of role to all age groups. Considering the role of women changes with age, it is necessary to study them further by defining the roles that are most appropriate for each age group. According to previous studies, the number of roles had different effects on mental health by age groups [20]. The middle age group (45–50 years old) had the worst mental health with 0 roles, followed by 4–5 roles. In older adults (70–75 years old), mental health was the worst when they played the most roles (2 roles). Although it was not included in the results of this paper, a frequency analysis of the roles performed by age group showed that the type of role is different for each age group. In the 20s, 55.9% were workers, whereas the proportion of other roles was low. In the 30s and 40s, the number of parents and houseworkers increased significantly, and more than half performed all roles except as a caregiver. As the role performed by the different age groups was different, it is considered to be meaningful to conduct comparative studies by age. In addition, there was a limitation that women’s roles were defined only in the work and family domain in this study. Women participate in various roles away from the work and family sphere, such as leisure activities and political activities, and so on, as members of society [39,40]. Especially, leisure activities in Korean women have been sacrificed due to ideological conflicts between “modernization” and “good wife and wise mother” [41]. Therefore, it is important to broaden women’s roles away from work and the family sphere. Furthermore, these activities linked to social resources and support. Despite these reasons, this study did not include them. In later studies, it is necessary to further expand the role of women to discuss multiple roles from various perspectives.

Second, all other role groups tended to be less depressed compared with the (None) role group. This finding is consistent with previous studies, which showed differences in the extent of roles but that participants were more depressed when they had no social roles across all age groups [9]. Roles in the home and occupations represent individual social relationships. Women having multiple roles could be less depressed than those who played relatively a small number of roles because social support can be increased through children, spouses, or co-workers. Meanwhile, women who did not play any role may reflect a group whose social relations are broken. Working, doing housework, getting married, rearing children and grandchildren, and caring for the family are commonly experienced by everyone during a lifetime, but performing no roles might prove that they had other problems. However, the role of houseworker was defined as having housework labor time that exceeds the average; the role of student was not even included in the analysis. The group without any role might have roles, but the results did not appear so because of the definition for this analysis and limitations mentioned above. On the other hand, it was difficult to find a specific arrangement in which the depressive patterns changed by adding specific roles or by increasing or decreasing the number of roles. The roles of housework, parenting, and spouse were mixed and difficult to separate clearly. This was similar to the result of a mixed and undivided housework and parenting in a study that classified subgroups based on tensions and benefits of women’s multiple roles [42]. In an additional cluster analysis to classify role groups in this study, they were classified into three groups: (1) worker role, (2) caregiver role, and (3) family role: spouse, parent, and houseworker. There were limitations to combining them into one role, as each role has its own characteristics. The number of single-parent families is rising and proportion of women has been increasing that get support or help for housework and childcare from family or professionals so that they can focus on their careers. Therefore, finding and applying criteria that can clearly distinguish home based roles are necessary.

Third, the caregiver role showed a negative effect on depression, which was in contrast with the result that other roles had positive effects on depression. Preliminary studies on family care and depression have shown that women were more likely to care for their families, and about half of family caregivers were depressed [43,44,45]. The tendency of caring assigned to women can be interpreted as the role of caregiver can be a burden for women. Previous studies have shown that caregiving was a burden on caregivers and had a negative effect on depression [45,46]. The role of caregiver had a negative effect on depressive symptoms when other variables, such as other roles, socioeconomic characteristics, and health status, were adjusted. It can be interpreted that the positive effects of performing other roles do not relieve the tensions that occur in the caregiving role. Although family care involves caring for not only parents but also children or spouses, there was a limitation in that participants who perform such family care were not included in the study because panel data were used. Moreover, the number of participants performing the role of caregiver was smaller than the total number of participants. Therefore, there was a limit to categorize them as “other” in the analysis of the role combination. The negative association between the role of caregiver and depression indicates that there is a possibility of different results if the role is included as a separate category in the role combination analysis. Therefore, further studies will be required to identify the positive and negative transitions of multi-role, focusing on participants with the caregiver role. Although there are leave policies for a family caregiver in Korea, it is necessary to make more policies to support various aspects for them, such as mental health management, financial support, and so on.

## 5. Conclusions

This study illustrated that depressive symptoms were influenced by not the number of roles but the combination of different types of roles. Among women who performed multiple roles, the group of women who did not perform any social role, performed the caregiver role, and performed a lower number of roles were most vulnerable to depressive symptoms. Moreover, role-combination was a more meaningful element in depressive symptoms of women.

The significance of this study was to identify the various aspects of women’s roles in determining the effects of multiple roles on depressive symptoms in women. The parent role was not simply defined as raising children but extended to include the concept of caring for grandchildren. Moreover, the effects of various role combinations on depressive symptoms were identified, and the role groups requiring appropriate intervention were selected and presented.

Despite the significance of this study, it has certain limitations. First, the role of caregivers expanded women’s multiple roles, but the role combination analysis did not lead to meaningful results in the role combination analysis because of the small sample size. Moreover, because of the limitations in using panel data, the role of caregivers was limited to caring for parents or parents-in-law. Second, there was a limit to fully capturing the changes in the roles of women with the life cycle. Therefore, considering the changes in women’s roles, we suggest that future studies must identify the effects of multiple roles on depression by applying different role groups for each age group.

## Figures and Tables

**Table 1:** Socio-demographic and role-related characteristics (N = 6198).

		N	%
		Mean (Range)	SD
Age (years)		53.01 (20–79)	11.3
	20s	37	0.6
	30s	711	11.5
	40s	1863	30.0
	50s	1547	25.0
	≥60	2040	32.9
Education	≤Elementary school	1408	22.7
	Middle school	818	13.2
	High school	2229	36.0
	≥College	1743	28.1
Income (×10^2^ KRW)	Q1 (≤2000)	1606	25.9
	Q2 (2001–4000)	1758	28.4
	Q3 (4001–6000)	1535	24.8
	Q4 (≥6001)	1299	21.0
Average of Annual Income (KRW)	4249.35 (0–35,360)	3070.1	
Marital Status	Unmarried	116	1.9
	Married	5150	83.1
	Separated	31	0.5
	Divorced	251	4.0
	Bereaved	650	10.5
Diagnosed Disease	No	4352	70.2
	Yes	1846	29.8
Number of Diagnosed Disease	0	4352	70.2
	1	1497	24.2
	2	291	4.7
	3	52	0.8
	4	5	0.1
	5	1	0.0
CES-D10 Score	4.35 (0–30)	4.92	
Worker role	No	2446	39.5
	Yes	3752	60.5
Houseworker role	No	2974	48.0
	Yes	3224	52.0
Parent role	No	3675	59.3
	Yes	2523	40.7
Spouse role	No	1048	16.9
	Yes	5150	83.1
Caregiver role	No	6132	98.9
	Yes	66	1.1
Number of Role	0	242	3.9
	1	1021	16.5
	2	1939	31.3
	3	2171	35.0
	4	822	13.3
	5	3	0.0
Combination of Role ^1^	None	242	3.9
	P-S	323	5.2
	W	376	6.1
	S-H	487	7.9
	S	525	8.5
	P-W-S	542	8.7
	P-S-H	705	11.4
	P-W-S-H	798	12.9
	W-S	853	13.8
	W-S-H	866	14.0
	Etc.	481	7.8

^1^ W: Worker role, H: Houseworker role, P: Parent role, S: Spouse role, C: Caregiver role.

**Table 2:** Effects of role fulfillment on depressive symptoms.

	B	SE	β	T	*p*-Value
Age	−0.003	0.009	−0.007	−0.318	0.751
Income	−0.396	0.069	−0.087	−5.712	0.000
Education					
≤Elementary school	Ref.				
Middle school	−0.204	0.207	−0.014	−0.986	0.324
High school	−0.215	0.206	−0.021	−1.048	0.295
≥College	−0.485	0.245	−0.044	−1.981	0.048
Diagnosed Disease (ref. no)	2.662	0.148	0.247	17.957	0.000
Worker role (ref. no)	−0.521	0.122	−0.052	−4.264	0.000
Houseworker role (ref. no)	−1.611	0.169	−0.123	−9.561	0.000
Parent role (ref. no)	0.082	0.162	0.008	0.506	0.613
Spouse role (ref. no)	−0.673	0.119	−0.068	−5.64	0.000
Caregiver role (ref. no)	1.379	0.565	0.029	2.439	0.015
R-squared	0.143				
F	93.749				
*p*	0.000				

**Table 3:** Effects of the number of roles on depressive symptoms.

	B	SE	β	T	*p*-Value
Age	−0.02	0.008	−0.045	−2.409	0.016
income	−0.474	0.069	−0.104	−6.897	0.000
Education					
≤Elementary school	Ref.				
Middle school	−0.303	0.207	−0.021	−1.465	0.143
High school	−0.27	0.206	−0.026	−1.31	0.190
≥College	−0.49	0.245	−0.045	−2.002	0.045
Diagnosed Disease (ref. no)	2.678	0.148	0.249	18.056	0.000
Number of Role	−0.647	0.067	−0.136	−9.614	0.000
R-squared	0.134				
F	137.132				
*p*	0.000				

**Table 4:** Effects of the combination of role on depressive symptoms.

	B	SE	β	T	*p*-Value
Age	−0.01	0.009	−0.023	−1.105	0.269
Income	−0.418	0.069	−0.092	−6.039	0.000
Education					
≤Elementary school	ref.				
Middle school	−0.218	0.207	−0.015	−1.054	0.292
High school	−0.225	0.207	−0.022	−1.088	0.277
≥College	−0.493	0.246	−0.045	−2.003	0.045
Diagnosed Disease (ref. no)	2.629	0.149	0.244	17.700	0.000
Combination of Role ^1^					
None	ref.				
P-S	−2.371	0.418	−0.107	−5.674	0.000
W	−1.751	0.381	−0.085	−4.596	0.000
S-H	−2.855	0.364	−0.156	−7.844	0.000
S	−2.181	0.358	−0.123	−6.088	0.000
P-W-S	−2.578	0.39	−0.148	−6.614	0.000
P-S-H	−3.324	0.378	−0.214	−8.783	0.000
P-W-S-H	−3.305	0.374	−0.225	−8.843	0.000
W-S	−2.839	0.342	−0.199	−8.302	0.000
W-S-H	−3.351	0.342	−0.236	−9.802	0.000
Others	−1.323	0.364	−0.072	−3.639	0.000
R-squared	0.144				
F	65.124				
*p*	0.000				

^1^ W: Worker role, H: Houseworker role, P: Parenting role, S: Spouse role, C: Caregiver role.
